# Bacteremia evaluation with T2 magnetic resonance in the emergency room (BETTER)

**DOI:** 10.1128/spectrum.03617-25

**Published:** 2026-07-15

**Authors:** Anna Maria Peri, Jaimi H. Greenslade, Angela Z. Hills, Mercedes T. Ray, Patrick N. A. Harris, David L. Paterson, Julian M. Williams

**Affiliations:** 1 UQ Centre for Clinical Research, The University of Queensland 1974, Brisbane, Queensland, Australia; 2 Herston Infectious Diseases Institute, Brisbane, Queensland, Australia; 3 Infectious Diseases Unit, Royal Brisbane and Women’s Hospital, Brisbane, Australia; 4 Emergency and Trauma Centre, Royal Brisbane and Women’s Hospital, Brisbane, Australia; 5 Faculty of Health, Queensland University of Technology 1969, Brisbane, Australia; 6 Central Microbiology, Pathology Queensland, Royal Brisbane and Women’s Hospital, Brisbane, Queensland, Australia; 7 ADVANCE-ID, Saw Swee Hock School of Public Health, National University of Singapore 37580, Singapore, Singapore; 8 Infectious Diseases Translational Research Programme, Yong Loo Lin School of Medicine, National University of Singapore 37580, Singapore, Singapore; 9 Faculty of Medicine, University of Queensland 1974, Brisbane, Australia; Assistance Publique - Hopitaux de Paris Universite Paris Saclay, Clamart, France

**Keywords:** rapid diagnostic test, blood culture, bloodstream infection, sepsis

## Abstract

Conventional blood culture (BC) is limited by long turnaround times and low sensitivity. The T2Bacteria is a novel culture-independent system identifying six bacteria from whole blood within a few hours. We conducted an observational study in an Australian Emergency Department to assess the performance of T2Bacteria, including its diagnostic accuracy, time to results, and potential role in early adjustment of antimicrobials. Patients with suspected bacteremia were enrolled and had BC drawn with T2Bacteria samples. We enrolled 101 patients. Thirty-five patients had positive BC, of which 31 (88.5%) grew T2Bacteria on-panel pathogens. Eight patients (26%) grew T2Bacteria on-panel pathogens in BC but had negative T2Bacteria results. Agreement between T2Bacteria and BC for T2Bacteria on-panel pathogens was 87.6%, sensitivity was 70.4%, and specificity 94.3%. Median time to results for T2Bacteria was 8.7 h, IQR 7.2–9.6, significantly shorter than time to Gram stain (17.0 h, IQR 12.2–21.6, *P* < 0.001) and MALDI-TOF MS (27.6 h, IQR 22.2–36.7, *P* < 0.001). Out of 19 patients with concordant T2Bacteria/BC results, T2Bacteria could have supported early antimicrobial de-escalation in seven cases (37%) and escalation in four cases (21%), including one case where antibiotics were not started based on suspicion of viral etiology, and three cases where the bloodstream pathogens were not covered by empirical antibiotics (*S. aureus*, *n* = 1, *P. aeruginosa*, *n* = 1, *E. faecium, n* = 1). T2Bacteria may support early escalation of antimicrobials in patients with suspected sepsis. Conversely, its narrow diagnostic panel and imperfect sensitivity do not support its use for early de-escalation.

**IMPORTANCE:**

This study evaluates the performance of T2Bacteria, a novel culture-independent test detecting six bloodstream pathogens directly from whole blood in 3–9 h, in patients with suspected sepsis admitted to the Emergency Department of an Australian hospital. Despite limitations in the diagnostic accuracy of T2Bacteria, in particular in its sensitivity, with some bacteremia episodes missed, we confirmed a much shorter turnaround time of T2Bacteria compared with conventional blood culture. These characteristics could support the use of T2Bacteria for early escalation of antimicrobials in cases of detection of pathogens not covered by empirical antibiotics, potentially reducing adverse clinical outcomes associated with exposure to ineffective antimicrobial regimens. Conversely, improved diagnostic performance and a broader diagnostic panel are likely required to recommend the use of T2Bacteria for early de-escalation. In our cohort, T2Bacteria supported early escalation in 4/19 cases with concordant BC and T2Bacteria results.

## INTRODUCTION

Sepsis and septic shock are life-threatening conditions caused by invasive micro-organisms and consequent immune and inflammatory responses (1). One of the critical early investigations indicated for emergency department (ED) patients with sepsis is blood culture (BC), the gold-standard test for identifying causative micro-organisms in the bloodstream. This enables targeted antimicrobial therapy, ensuring pathogens are eradicated, abating deleterious inflammatory responses, and decreasing associated mortality (2, 3).

Current methods of BC collection, processing, and analysis have changed little in the last 50 years. Actionable results are not available to guide initial ED workup, and final results may not be available for several days (4). Moreover, BC-based methods are known to have limited sensitivity, particularly in cases of recent exposure to antimicrobials, potentially leading to “culture-negative” sepsis episodes (5, 6). As a result, many patients remain on broad-spectrum antibiotics for extended courses. This is associated with the development of multidrug-resistant bacteria, drug side effects, and complications such as antibiotic-associated colitis (7).

Developing alternative means for identifying pathogenic bacteria in the bloodstream is an active field of research with some promising developments (8). However, most emerging methods rely on BCs to initially amplify bacterial numbers in the blood before analysis, a step that usually takes 8-12 hours (9).

The T2 magnetic resonance system (T2 Biosystems, Lexington, MA, USA) uses PCR amplification and magnetic resonance-based molecular diagnostic technology to identify selected pathogenic microbial elements directly from whole blood, with results reportedly available in 3–9 h (10). The T2Bacteria (T2B) panel detects six pathogenic bacteria—*Staphylococcus aureus, Escherichia coli, Klebsiella pneumoniae, Pseudomonas aeruginosa, Enterococcus faecium*, and *Acinetobacter baumannii*—which collectively account for 50%–60% of pathogens reported from BCs taken from patients in Australian EDs (11, 12). Some studies have shown T2 magnetic resonance more sensitive than BC, particularly in patients exposed to antimicrobials (13, 14).

Although early studies examining the clinical performance of T2B provided encouraging results (15), further data are required to establish accuracy and clinical utility. Several studies have been limited by cohorts with low prevalence of positive BCs and low-quality comparator BC techniques (16). To date, no data have been gathered in the Australian ED setting. The aim of this study was to assess the performance of the novel T2B panel for detection of bacteremia in ED patients at high risk for bloodstream infection.

## MATERIALS AND METHODS

### Setting

This prospective observational study was conducted in the ED of an Australian urban tertiary referral hospital treating 85,000–90,000 adult presentations annually. Patient enrollment occurred between February 2023 and February 2024. Approval was granted by the local ethics committee (HREC/2023/MNHA/94258) and ratified by the University of Queensland (2025/HE000780). All recruited subjects or their representatives consented to participation.

### Aims

Specific study questions were as follows:

1. What is the diagnostic accuracy of T2B in ED patients with suspected bloodstream infection and hypoperfusion?
2. In BC-negative / T2B-positive (BC−/T2B+) cases, what is the clinical evidence for infection with T2B-identified pathogens?
3. In cases with positive BC and positive T2B results, what was the time difference to result?
4. In cases with positive BC and positive T2B results, would T2B have supported early adjustment of antimicrobials?

### Subjects

Adult patients aged >18 years old presenting to the ED with suspected bloodstream infection and hypoperfusion (systolic blood pressure [SBP] <90 mmHg or lactate ≥4.0 mmol/L) were eligible for enrollment. Investigation and treatment of these patients proceeded as per departmental guidelines based on current evidence (17), including two sets of BCs, other cultures as appropriate, organ support interventions, and antimicrobial therapy. Based on prior work, 35% of patients enrolled in the study were expected to have positive BCs, and in total 55% were expected to have any positive microbiological test (18). The study sample size of 100 patients was determined by available funding for T2B analysis.

### Measurements

BCs were collected in BacT/Alert FA PLUS and FN PLUS bottles and incubated with the BacT/Alert Virtuo (bioMérieux, France) system for up to 5 days. Samples from positive culture bottles were Gram stained, plated, and further incubated for a maximum of 48 h. BCs were plated on blood, chocolate, and MacConkey agar. In the case of growth from the anaerobic bottle, anaerobic media were additionally inoculated. Additionally, one drop from positive BCs was applied to a chocolate agar plate and incubated for 4–6 h (CHOC spot). Pathogens growing on plates (including from the CHOC spot) underwent species identification with matrix-assisted laser desorption/ionization time-of-flight mass spectrometry (MALDI-TOF MS) (VITEK MS, bioMerieux, France) and susceptibility testing with VITEK 2 (bioMerieux, France). For each positive BC, data collected included time to Gram stain result and time to pathogen identification with MALDI-TOF MS.

Samples for T2B analysis were drawn in EDTA tubes and frozen at −70°C according to the freezing procedure allowed by the T2B package insert (19). After recruitment of 101 study patients, samples were thawed and retrospectively batch analyzed, otherwise as per manufacturer specifications. Specifically, 2 mL of whole blood from each sample was pipetted into the T2B cartridge. Time to result was recorded for each T2B sample.

For each patient, clinical and microbiological data were abstracted from hospital records, databases of hematology, biochemistry, and microbiological results, and imaging as relevant. Times of antimicrobial administration, BC collection, and T2B sample collection were recorded. BC contamination was defined according to standard definitions (20).

### Analyses

Analyses were performed using Stata, version 17 (StataCorp LLC). Baseline continuous variables were presented as mean (SD) or median (IQR), and categorical variables as *n* (%). Categorical variables were compared using Fisher’s exact test or Chi-squared test as appropriate. Continuous variables were compared using the Wilcoxon rank-sum test. Diagnostic accuracy statistics (sensitivity, specificity, positive predictive value [PPV], and negative predictive value [NPV]) were calculated with 95% confidence intervals.

The potential role of T2B in supporting early adjustment of antimicrobials was assessed. To this respect, de-escalation was broadly defined as any potential change of empirical antimicrobials to remove unnecessary coverage based on T2B results (i.e., removal of Gram-positive coverage in the case of identification of Gram-negative pathogens and vice versa). Similarly, escalation was defined as any potential change of antimicrobials to add coverage of pathogens identified by the T2B against which empirical therapy was not effective (e.g., introduction of vancomycin in the case of detection of *E. faecium* or introduction of anti-pseudomonal therapy in the case of detection of *P. aeruginosa* if these pathogens were not covered by empirical regimens, respectively). Escalation and de-escalation were not defined based on suppositions on the susceptibility profile of the specific species identified (e.g., switch from piperacillin-tazobactam to ampicillin in the case of detection of *E. coli*).

## RESULTS

### Patients’ characteristics and T2B diagnostic accuracy

There were 101 patients enrolled in the study; clinical characteristics are detailed in Table 1. Males comprised 56%, and median age was 69 years (IQR 57–78 years). The most common sources of infection were respiratory (32.7%) and urinary tract (23.8%). Eighty-nine patients (88.1%) had two sets of BCs collected, while 12 patients (11.9%) had one set of BCs collected. After excluding contaminants, BCs were positive in 35 (34.7%) patients. Of these, five had polymicrobial growth, accounting for a total of 40 isolates grown according to conventional BC methods. The T2B assay identified 28 pathogens from 23 (22.8%) patients, returned an invalid result in one case (failure of internal control), and failed to provide a result due to machine dysfunction in a further three cases. Fig. S1 shows the isolates grown from BCs with *E. coli* (*n* = 12), *S. aureus* (*n* = 7), *P. aeruginosa* (*n* = 6), and *K. pneumoniae* (*n* = 5) being the most commonly identified isolates. One BC was positive for *E. faecium*. Overall, isolates included on the T2B panel comprised 31/40 (77.5%) of all isolates grown on BC.

**TABLE 1:** Clinical characteristics of study patients^*a*^

Variable	All patients *n* = 101
Demographics	
Age in years, median (IQR)	69 (57–78)
Male, *n* (%)	57 (56.4%)
Nursing home residence, *n* (%)	6 (5.9%)
Morbidities, *n* (%)	
Diabetes	29 (28.7%)
Renal failure	3 (3.0%)
Ischemicc heart disease	12 (11.9%)
Heart failure	13 (12.9%)
Chronic lung disease	34 (33.7%)
Charlson score, median (IQR)	6 (3-8)
ED measurements, *n* (%)	
Systolic blood pressure <90 mmHg	84 (83.2%)
Lactate ≥4.0 mmol/L	34 (34%)
Temperature <36.0°C or ≥38.0°C	51 (50.5%)
Respiratory rate >20 breaths/minute	77 (76.2%)
Heart rate >90 beats/minute	82 (81.2%)
White cell count <4 or >12 × 10^9^/L	57 (56.4%)
New confusion (GCS reduced from baseline)	29 (28.7%)
Organ dysfunction (SOFA ≥2)	76 (75.2%)
Suspected source of infection, *n* (%)	
Urinary tract	24 (23.8%)
Respiratory	33 (32.7%)
Skin	13 (12.9%)
Intra-abdominal	10 (9.9%)
Unknown/primarybacteremia	13 (12.9%)
Interventions	
Blood cultures ≥2 sets, *n* (%)	89 (88.1%)
Time to effective antibiotics^*b*^, hours, median (IQR)	1.9 (1.0–3.5)
Noradrenaline use, *n* (%)	25 (24.8%)
Intensive care admission, *n* (%)	24 (23.8%)
Outcomes	
Bacteremia (positive BC), *n* (%)	35 (34.7%)
Any positive microbiological results (samples taken within 48 h of arrival), *n* (%)	61 (60.4%)
T2Bacteria positive, *n* (%)	23 (22.8%)
Hospital length of stay, days, median (IQR)	6 (4–9)
30-day mortality, *n* (%)	12 (11.9%)

^*a*^ Two patients did not have lactate measured, and six patients did not receive an effective antibiotic in the emergency department.

^*b*^ Effective antibiotics were defined as those to which isolated micro-organisms demonstrated sensitivity in culture positive cases, and those consistent with empiric guidelines in culture negative patients.

**Fig 1:**
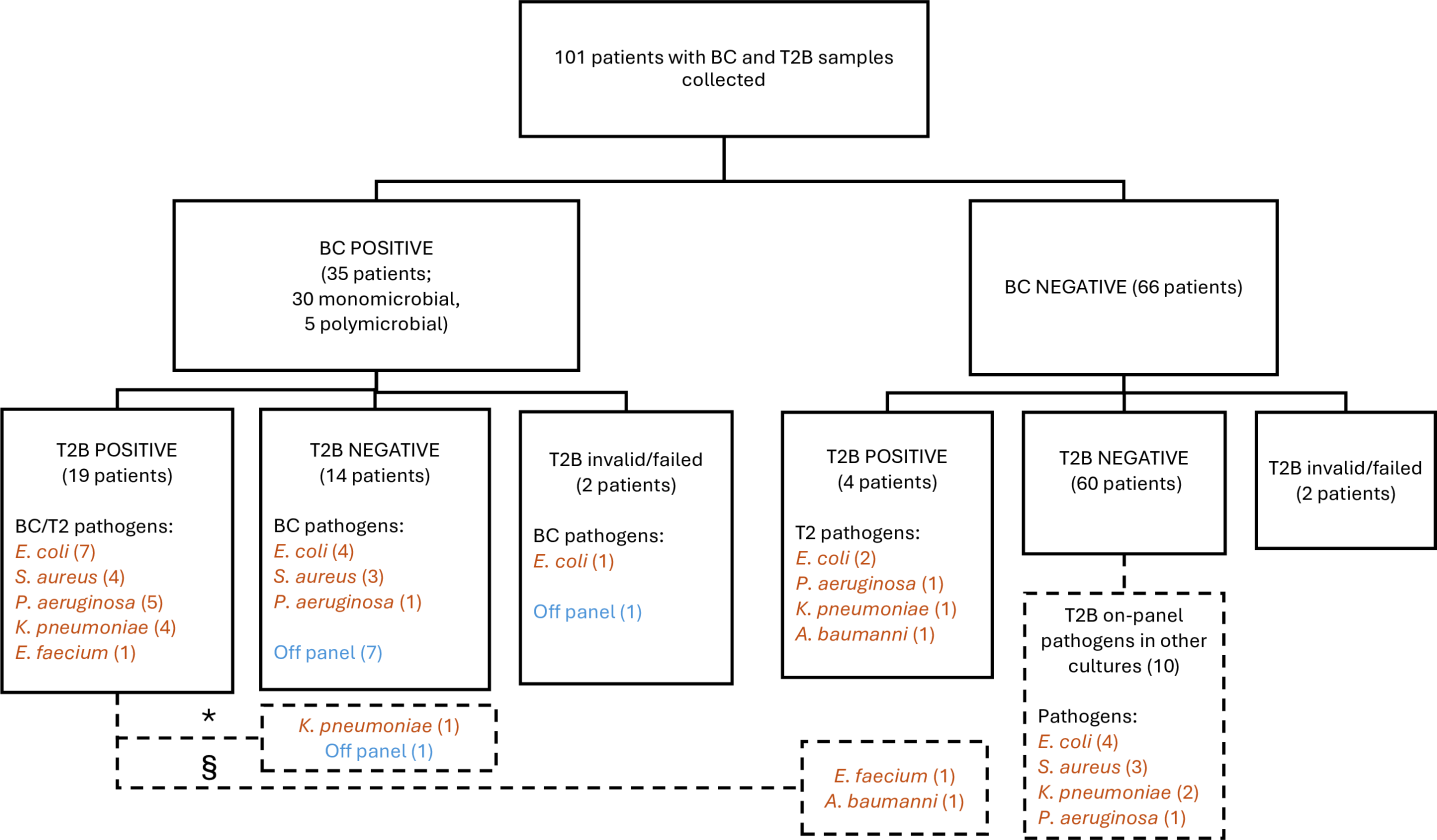
Study patients, blood culture, and T2Bacteria results. *Two patients had additional BC isolates not demonstrated with T2Bacteria, § two patients had additional T2Bacteria isolates not demonstrated with blood culture. BC = blood culture; T2B = T2Bacteria result.

Figure 1 and Fig. S2 show BC and T2B results. Nineteen patients had pathogens identified by both BC and T2B (BC+/T2B+), and 15 had identical pathogens on both panels. The remaining four patients had a concordant pathogen detected by BC and T2B plus an additional pathogen detected by either of the two tests (two by BC and two by T2B). Fourteen patients grew bacteria on BC but were T2B negative (BC+/T2B−, 14%). Eight of these patients grew T2B on-panel bacteria in BC (8%). BCs were negative in four patients where the T2B identified organisms (BC−/T2B+, 4%). At the patient level, agreement between T2B and BC when considering all pathogens was 81.4%, with sensitivity 57.6% and specificity 93.8%. If considering only T2B on-panel pathogens, agreement was 87.6%, sensitivity was 70.4%, and specificity was 94.3%. Table 2 reports diagnostic accuracy measures for the T2B.

**TABLE 2:** Diagnostic accuracy for all bacteria and T2Bacteria on-panel bacteria^*b*^

All bacteria (patients)			
	BC pos (35)	BC neg (66)	Diagnostic accuracy^***a***^
T2B positive	19	4	Sensitivity: 57.6% (95% CI: 39.2%–74.5%) Specificity: 93.8% (95% CI: 84.8%–98.3%) PPV: 82.6% (95% CI: 61.2% to 95.0%) NPV: 81.1% (95% CI: 70.3%–89.3%).
T2B negative	14	60	
T2B invalid/failed runs	2	2	

T2B on-panel bacteria (patients)			
	BC pos (28)	BC neg (73)	Diagnostic accuracy^***a***^
T2B positive	19	4	Sensitivity 70.4% (95% CI: 49.8%–86.2%) Specificity 94.3% (95% CI: 86.0%–98.4%) PPV 82.6% (95% CI: 61.2%–95.0%) NPV 89.2% (95% CI: 79.8%–95.2%)
T2B negative	8	66	
T2B invalid/failed runs	1	3	

All bacteria (pathogens)			
	BC pos (40)	BC neg (69)	Diagnostic accuracy^***a***^
T2B positive (28)	21	7	Sensitivity 55.3% (95% CI: 38.2%–71.4%) Specificity 89.6% (95% CI: 79.7%–95.7%) PPV 75.0% (95% CI: 55.1%–89.3%) NPV 77.9% (95% CI: 67.0%–86.6%)
T2B negative (77)	17	60	
T2B invalid/failed runs (4)	2	2	

T2B on-panel bacteria (pathogens)			
	BC pos (31)	BC neg (78)	Diagnostic accuracy^***a***^
T2B positive (28)	21	7	Sensitivity 70.0% (95% CI: 50.6%–85.3%) Specificity 90.7% (95% CI: 81.7%–96.2%) PPV 75.0% (95% CI: 55.1%–89.3%) NPV 88.3% (95% CI: 79.0%–94.5%)
T2B negative (77)	9	68	
T2B invalid/failed runs (4)	1	3	

^*a*^ Excludes the four patients with invalid/failed runs.

^*b*^ BC, blood culture; T2B, T2Bacteria result.

An additional 26 patients (25.7%) demonstrated positive microbiology results on specimens other than blood. In 10 of these cases, identified micro-organisms were T2B on-panel bacteria; however, corresponding T2B tests were negative. One patient had an operative specimen culture positive for *E. faecalis* with T2B result positive for *A. baumannii*.

Table S1 summarizes clinical information of patients with T2B+/BC− results (*n* = 6). Of these, two were deemed to be T2B true positives, while four were deemed to be T2B false positives according to clinical adjudication. Among the ones deemed T2B true positives, there was one patient admitted for cholangitis managed conservatively with T2B positive for *E. coli*. The patient responded to antibiotic therapy (ceftriaxone and metronidazole) but was readmitted a month later for relapse of biliary sepsis, this time with a BC-proven bacteremia due to *E. coli*. The second case deemed as a T2B true positive was of a patient admitted for cellulitis with T2B positive for *P. aeruginosa*. The patient responded to antibiotic course but after discharge was readmitted for two distinct episodes of BC-proven bacteremia due to *P. aeruginosa*, 2 and 7 months after the index admission, respectively. The remaining four cases were deemed as T2B false positives. Among there was one patient admitted for suspected lower respiratory tract infection and T2B positive for *E. coli*; a patient admitted for necrotizing fasciitis of the leg with T2B positive for *A. baumannii* (but intraoperative specimens positive for *E. faecalis*); a patient with colitis and BC-proven bacteremia due to *K. pneumoniae* with T2B also positive for *E. faecium,* who responded to piperacillin-tazobactam; and a bone marrow transplant patient with a BC-proven bacteremia due to *K. pneumoniae* from urinary source where T2B was positive for *A. baumannii* as well.

### Timing of sample collection and antibiotic administration

Antimicrobials had been administered to 49/101 patients (48.5%) prior to blood collection for T2B analysis. Of these T2B samples, 11/49 (22.4%) returned a positive result. Of the 52/101 (51.5%) patients who did not have antimicrobials administered prior to T2B blood collection, 12 (23.1%) returned a positive T2B result (*P* = 0.8). Moreover, antibiotics were administered to 11/101 (10.9%) patients prior to BC collection; of these, three had positive BC (27%), of which two also had positive T2B. The third patient had positive BC only (with negative T2B); of note, this last patient had his T2B sample collected post-antibiotics. All BC−/T2B + patients (Table S1) had antibiotics administered after both BC and T2B sample collection.

When comparing patients who had antimicrobials administered before T2B sample collection but after BC collection (*n* = 40) vs patients who had antimicrobials administered after both BC and T2B collection (*n* = 50), no significant differences between T2B and BC positivity rates were found (i.e., BC+/T2B− 5/40, 12.5% vs 2/50, 4%, *P* = 0.2; BC+/T2B + 9/40, 22.5% vs 8/50, 16% [*P* = 0.6; BC-/T2B + 0/40, 0% vs 4/50, 8%, *P* = 0.13).

Overall, 56 patients had T2B and BC samples collected within 1 h, and 45 had T2B and BC samples collected more than 1 h apart (median 2.2 h, IQR 1.2–1.8). No significant differences were noted in BC and T2B positivity rates among patients who had T2B and BC samples drawn within an hour and those who had T2B and BC samples drawn more than 1 h apart (data not shown). Specifically, among four BC−/T2+patients, two had BC and T2B samples taken within 1 h and two taken more than 1 h apart. Similarly, out of 8 BC+/T2− patients, four had BC and T2B samples taken within 1 h and four taken more than 1 hour apart.

No BC−/T2B + patients were found among patients with only set of BCs drawn.

### Time to results

Time to results of Gram stain, MALDI TOF MS, and T2B are summarized in Table 3.

**TABLE 3:** Time to results of BCs and T2Bacteria^*a*^

	Time to gram stain (h)	Time to MALDI-TOF MS results (h)	Time to T2Bacteria results (h)	*P* value
All samples with a positive result, either BC or T2	17.0 (12.2–21.6)	27.6 h (22.2–36.7)	8.7 (7.2–9.6)	Gram stain vs T2Bacteria: *P* < 0.001 MALDI-TOF vs T2Bacteria: *P* < 0.001
Samples with concordant T2Bacteria and BC result	12.6 (11.5–17.9)	23.9 (22.0–30.8)	8.7 (7.2–9.5)	Gram stain vs T2Bacteria: *P* < 0.001 MALDI-TOF vs T2Bacteria: *P* < 0.001

^*a*^ All times are expressed in median (IQR).

Median time from BC collection to laboratory receipt was 37 min (IQR 24–78). This was added to the time to T2B results, creating a “theoretical time to result” that more accurately reflects delays experienced in clinical practice.

When including all pathogens grown on BC (*n* = 40), median time to Gram stain result was 17.0 h (IQR 12.2–21.6), while median time to identification based on MALDI-TOF MS was 27.6 h (IQR 22.2–36.7). When including all T2B positive tests (*n* = 23), median time to results was 8.7 h (IQR 7.2–9.6), significantly shorter than both time to Gram stain (*P* < 0.001) and MALDI-TOF MS identification (*P* < 0.001). When including only samples with a concordant BC and T2B results (*n* = 21), time to Gram stain was 12.6 h (IQR 11.5–17.9) while time to MALDI-TOF MS identification was 23.9 h (IQR 22.0–30.8), compared with 8.7 h (IQR 7.2–9.5) (*P* < 0.001) for T2B results.

For *S. aureus*, preliminary results based on coagulase testing were available before MALDI-TOF MS results. For samples positive for *S. aureus* with a concordant BC and T2B result (*n* = 4), time to T2B was 7.1 h (IQR 6.3–9.5), significantly shorter than time to identification based on coagulase testing, which was 15.6 h (IQR 12.4–19.4) (*P* = 0.021).

### Potential utility of T2B for early adjustment of antimicrobials

Table 4 reports empirical antibiotics administered before the hypothetical availability of the T2B results in patients with concordant T2B and BC results (*n* = 19), and the potential role of T2B in early adjustment of antimicrobials. Out of 19 patients with concordant T2B and BC results, T2B could have supported early de-escalation in seven cases (37%). Specifically, it would have supported early discontinuation of vancomycin in three cases where only Gram-negative pathogens were detected, early discontinuation of metronidazole in one case where *S. aureus* was detected*,* and early discontinuation of azithromycin in another case where *E. coli* was detected. In two additional cases, the early detection of *S. aureus* by T2B would have supported early adjustment of broad-spectrum empirical antimicrobials to targeted anti-staphylococcal agents.

**TABLE 4:** Empirical antibiotics administered before hypothetical availability of the T2Bacteria results in patients with concordant T2Bacteria and BC results (*n* = 19), and the potential role of T2Bacteria in early adjustment of antimicrobials

Potential role of T2B in early adjustment of antimicrobials	Pt ID	T2/BC concordant pathogen	T2/BC concordant pathogen 2	Empirical antibiotics, given before T2Bacteria results	How would have T2Bacteria helped early adjustment of antimicrobials?	Comments
De-escalation	5	*S. aureus*		Flucloxacillin, gentamicin, metronidazole	Stop metronidazole	
	18	*S. aureus*		Cefepime, gentamicin	Adjust to anti-staphylococcal therapy	
	43	*S. aureus*		Piperacillin-tazobactam, gentamicin, later switched to meropenem and vancomycin	Adjust to anti-staphylococcal therapy	
	62	*K. pneumoniae*		Piperacillin-tazobactam, gentamicin, later switched to meropenem and vancomycin	Stop vancomycin, possibly de-escalate meropenem	BC also grew *E. cloacae*
	81	*K. pneumoniae*	*P. aeruginosa*	Piperacillin-tazobactam, gentamicin, vancomycin	Cease vancomycin	
	90	*E. coli*		Ceftriaxone, azithromycin, gentamicin	Cease azithromycin	
	94	*K. pneumoniae*		Piperacillin-tazobactam later switched to meropenem, vancomycin	Cease vancomycin	Patient had *A. baumannii* also detected by T2B
Escalation	26	*K. pneumoniae*		None	Start antimicrobials	Patient had *E. faecium* also detected by T2B
	61	*S. aureus*		Ceftriaxone, azithromycin	Start anti-staphylococcal therapy, cease azithromycin	
	88	*P. aeruginosa*		Ampicillin, gentamicin	Start anti-pseudomonal therapy	
	95	*P. aeruginosa*	*E. faecium*	Piperacillin-tazobactam, gentamicin	Add vancomycin	
None	12	*E. coli*		Amoxicillin-clavulanate, gentamicin	No role, reasonable regimen for *E. coli,* awaiting sensitivities	
	25	*E. coli*		Ampicillin, gentamicin, metronidazole, later switched to piperacillin-tazobactam and metronidazole	No role, metronidazole was given in the suspect of *C. diff* colitis. Remaining regimen reasonable for *E. coli* awaiting sensitivities	
	29	*P. aeruginosa*		Piperacillin-tazobactam, gentamicin	No role, reasonable regimen for *P. aeruginosa* awaiting sensitivities	
	37	*E. coli*		Flucloxacillin, gentamicin, later switched to piperacillin-tazobactam	No role, piperacillin-tazobactam reasonable for *E. coli* awaiting sensitivities	
	48	*E. coli*		Piperacillin-tazobactam, gentamicin	No role, reasonable regimen for *E. coli* awaiting sensitivities	BC also grew *K. pneumoniae*
	66	*E. coli*		Ampicillin, gentamicin	No role, reasonable regimen for *E. coli* awaiting sensitivities	*E. coli* later found to be ESBL
	76	*P. aeruginosa*		Piperacillin-tazobactam	No role, reasonable regimen for *P. aeruginosa* awaiting sensitivities	
	89	*E. coli*		Piperacillin-tazobactam, gentamicin, later switched to amoxicillin-clavulanate	No role, reasonable regimen for *E. coli* awaiting sensitivities	

In 4/19 (21%) cases, T2B would have supported early escalation of antimicrobials, including one case where antibiotics were not started based on suspicion of viral etiology, and three cases where the bloodstream pathogens were not covered by empirical antibiotics (including *S. aureus, n* = 1, *P. aeruginosa*, *n* = 1, and *E. faecium, n* = 1).

In the remaining 8/19 cases (42%), early T2B results would not have resulted in any significant changes to administered therapy. In one of these eight cases, further escalation would later be needed based on antimicrobial susceptibility testing.

Overall, T2B results could have been useful for early adjustment of antimicrobials in 31% (11/35) of those patients with positive BC results.

Of note, in 2 of the 19 patients with concordant T2B and BC results, T2B detected an additional pathogen, not grown on BC, possibly interpretable as a false-positive result (*A. baumannii* and *E. faecium*). Early T2B-directed antimicrobial therapy would have likely resulted in excessively broad antimicrobial therapy in one case (addition of vancomycin to cover *E. faecium*, not grown on BC), while it would have likely not resulted in any changes in the other case, as the additional T2B pathogen (*A. baumannii*) was already covered by the empirical therapy administered (meropenem).

Additionally, in two further cases, T2B was able to detect one pathogen only from polymicrobial BCs, which grew two pathogens. In both cases, the pathogen missed by T2B (*K. pneumoniae* and *E. cloacae*) was reasonably covered by the empirical antimicrobial regimen.

## DISCUSSION

Our study investigated the performance and potential of the T2B panel in an Australian ED. As anticipated based on previous work (18), 35% (35/101) of patients enrolled in the study had positive BCs, potentially representing the target patient population of novel rapid diagnostic tests.

In our study population, T2B on-panel pathogens accounted for 77.5% (31/40) of bloodstream infection isolates. This is a coverage rate higher than that expected based on available literature on the epidemiology of bloodstream infection in Australian EDs (11, 12), as well as studies assessing T2B in non-exclusively critically ill patients (16, 21, 22), where the T2B coverage was around 48%–63%. Nonetheless, previous evaluations in critically ill patients with sepsis and septic shock showed a coverage of bloodstream isolates by T2B up to 81%–88%, more in line with our findings (23–25).

For T2B on-panel bloodstream infection episodes, we reported a specificity of 94.3% in line with previous studies (16, 23, 26). Conversely, the sensitivity of T2B (70.4%) was slightly lower than the 86%–100% previously demonstrated (16, 23, 24, 26–29). This difference might be explained by our gold standard comparator based on the use of two BC sets in most patients (88%), while other studies had used one set of BCs only (16, 24) or at least one set (23). A recent evaluation, however, showed T2B sensitivity of 100% when using two BC sets as comparator (26). The use of frozen rather than fresh blood samples may also have impacted sensitivity, though this is at odds with a previous finding (16).

The rate of BC−/T2B + samples reported by previous studies is variable, ranging from 2 to 20% (16, 22–24, 26–28, 30, 31), but overall, mostly >10% (16, 23, 26–28, 31). In our evaluation, T2B detected a pathogen not grown in the paired BC in 6% of cases (*n* = 6). The interpretation of these discrepant cases is not always straightforward based on the clinical information available. In our study, 2/6 patients with BC-/T2B + results were admitted for a BC-proven bacteremia due to the pathogen originally detected by the T2B a few weeks after the index admission, suggesting how T2B had likely detected a true pathogen. One of these patients, in particular, had an uncontrolled and persistent source of infection (cholangitis), which further supports the likelihood of the *E. coli* originally detected by T2B being a true positive. In the remaining 4/6 cases, instead, the T2B-positive results accompanying a negative BC were interpreted as false positives. One of these patients had a T2B positive for *A. baumannii,* which is a known target potentially giving false positive results as reported in the T2B package insert as well as in the literature (26).

Median time to T2B results was significantly shorter than time to Gram stain and identification (MALDI-TOF MS) of BC pathogens. Previous evaluations had reported shorter pathogen identification based on T2B vs MALDI-TOF MS (22–24, 26, 27, 30–32), but most had not made any comparisons between time to T2B and time to Gram stain, which is normally the first information available to clinicians. With respect to our reported time to results, we must acknowledge that we compared theoretical times to T2B results vs actual time to results of BCs. This could introduce bias, as our times to T2B results do not account for potential delays in running the test that could take place in the real-life setting of a clinical laboratory. Nonetheless, it is more likely that relying on theoretical times of batch testing would result in longer times than those that would be observed in a real-life scenario. Indeed, when running batched samples (filling all the seven drawers of the machine), the overall time to result of the instrument is longer compared with when a single sample is run (up to 9 h in the first case versus 3.5–4 h in the latter). In a real-life scenario where T2B testing was to be used only in septic patients from the ED, such as those included in this evaluation, it would be much more likely that samples were run one at a time, resulting in shorter turnaround times than those we reported.

Very few studies have so far evaluated the impact of T2B in early adjustment of antimicrobials (23, 27, 30, 31). In our evaluation, out of 19 patients with concordant T2B and BC results, we estimated that early T2B results may have been useful to adjust empirical therapy in 11/19 (58%) cases (overall representing 31% of all patients with confirmed bacteremia), including 7/19 (37%) de-escalations and 4/19 (21%) escalations. Nonetheless, the T2B panel is relatively narrow, and as demonstrated, its diagnostic accuracy is far from perfect. For these reasons, we would not recommend using T2B results to support early de-escalation of empirical antimicrobials, particularly in critically ill patients. T2B was, however, also useful to support escalation of antimicrobials in a subset of patients. Using T2B results to escalate antimicrobials could prevent patients from being exposed to ineffective empirical regimens. Given the adverse outcomes associated with exposure to ineffective antibiotics, using T2B results for antimicrobial escalation likely outweighs the risk that the patients may incur in the case of a false-positive T2B result, that is, being temporarily exposed to an excessively broad antimicrobial regimen.

This study has several limitations. First, the sample size was small, limiting the significance of the results. Second, as discussed, not all the patients had two sets of BCs drawn, introducing heterogeneity in the gold standard comparator. Third, T2B and BC samples were not always collected concurrently, and, in a few cases, antimicrobials were administered before T2B samples were collected, possibly affecting the performance of the assay. Fourth, this study was performed at a single center, limiting the generalizability of the results. Fifth, no analysis of cost-effectiveness or workflow burden was included. Finally, the impact of T2B on antimicrobial adjustments was just theoretical rather than based on actual changes.

In conclusion, well-designed randomized controlled trials (RCTs) are needed to confirm if early use of T2B results in improved clinical outcomes in patients with suspected sepsis. Health economic analyses are also needed to clarify the cost-effectiveness of introducing T2B in clinical practice, and the benefit of using rapid diagnostic tests incorporating the detection of antimicrobial resistance markers should also be evaluated. Importantly, T2B does not replace BC, and its results should be carefully interpreted within each specific clinical context.

## Data Availability

Data available upon authors’ request.

## SUPPLEMENTAL MATERIAL

Supplemental figures and table spectrum.03617-25-s0001.docx Figures S1 and S2; Table S1.

ASM does not own the copyrights to Supplemental Material that may be linked to, or accessed through, an article. The authors have granted ASM a non-exclusive, world-wide license to publish the Supplemental Material files. Please contact the corresponding author directly for reuse.
