# Supplementary material for: Bacteremia evaluation with T2 magnetic resonance in the emergency room (BETTER)
Source: Microbiol Spectr. 2026 Jul 15;14(8):e03617-25. doi: 10.1128/spectrum.03617-25 (PMC13435762; doi:10.1128/spectrum.03617-25)
Supplement: Supplementary file 1 [file spectrum.03617-25-s0001.docx]

**Supplementary figure 1:** Blood culture isolates (n=40) grown from 35 patients. T2Bacteria on-panel organisms are represented as pale blue, off-panel organisms are shown in dark blue.

MSSA = Methicillin-sensitive *Staphylococcus aureus.*

**Supplementary figure 2:** Study patients, blood culture and T2Bacteria results. BC = Blood Culture; T2B = T2Bacteria result

**Supplementary Table 1.** Clinical characteristics of patients with T2Bacteria positive / blood culture negative results.

| Pt n | T2B result | Paired BC result | Demographics, clinical presentation and antimicrobial course | Other relevant microbiology results | Clinical interpretation of T2B result |
| --- | --- | --- | --- | --- | --- |
| 40 | *E. coli*  *K. pneumoniae* | Negative | **Demographics:** 100-year-old, F  **Presenting complaint:** Abdominal pain and fever in recent cholangitis  **Suspected infection source and clinical course:** Biliary. Improved with conservative management and discharged but readmitted a month later for relapse of biliary sepsis with BC positive for *E. coli*  **Antibiotics:** Ceftriaxone, metronidazole for 6 days, discharged on metronidazole and trimethoprim-sulfamethoxazole for further 5 days. | *E. coli* in BCs collected 1 month post index episode, during readmission to hospital for relapse of biliary sepsis. | Likely true positive |
| 41 | *P. aeruginosa* | Negative | **Demographics:** 77-year-old, F  **Presenting complaint:** Left leg erythema and swelling in the context of septic shock  **Suspected infection source and clinical course:** Skin and soft tissue (left leg cellulitis). Cellulitis worsened during the initial course of flucloxacillin, leading to escalation to piperacillin-tazobactam (continued for 24 hours) and clindamycin. Full recovery after antibiotic change. Of note, the patient was readmitted to hospital for 2 distinct episodes of *P. aeruginosa* BSI occurred 2 months and 7 months after the index admission respectively.  **Antibiotics:** Flucloxacillin for 5 days, then piperacillin-tazobactam (for 24 hours only) and clindamycin (for further 5 days) | *P. aeruginosa* in BCs collected 2 months and 7 months after the index episode. Of these 2 BSI episodes the first was thought to have unknown source, the second was thought to originate from a recurrence of the left leg cellulitis | Likely true positive |
| 44 | *E. coli* | Negative | **Demographics:** 62-year-old, F  **Presenting complaint:** Syncope, increased shortness of breath, fever  **Suspected infection source and clinical course:** Respiratory (lower respiratory tract infection with no consolidation on chest X ray).  **Antibiotics:** Ceftriaxone and azithromycin for 2 days then amoxicillin for further 5 days | None | Likely false positive |
| 82 | *Acinetobacter* | Negative | **Demographics:** 75-year-old, M  **Presenting complaint:** Necrotic wound post skin excision of right calf lesion in the context of septic shock, on a background of myelodysplastic syndrome.  **Suspected infection source and clinical course:** Skin and soft tissue (right leg necrotizing fasciitis). Required multiple surgeries for debridement.  **Antibiotics:** Initial single dose of piperacillin-tazobactam, then escalated to meropenem, clindamycin and vancomycin for a ~2-week course. Subsequent step down to amoxicillin-clavulanate for further 10 days. | *E. faecalis* grown from operative specimen of leg wound. | Likely false positive |
| 26 | *K. pneumoniae* *E. faecium* | *K. pneumoniae* | **Demographics:** 75-year-old, M  **Presenting complaint:** Diarrhoea, vomit and fever in the setting of COVID-19 infection  **Suspected infection source and clinical course:** Abdominal. Diagnosed with colitis and bacteraemia due to *K. pneumoniae.*  **Antibiotics:** None in ED (presumed viral cause), then ceftriaxone and metronidazole for 1 day, subsequently adjusted to piperacillin-tazobactam for 8 days. | Reported to have had *S. aureus* in post operative wound prior to this presentation. | Likely false positive |
| 94 | *K. pneumoniae*  *Acinetobacter* | *K. pneumoniae* | **Demographics:** 42-year-old, M  **Presenting complaint:** Fever, cough and shortness of breath on a background of previous bone marrow transplant for acute myeloid leukaemia, cavitating pulmonary aspergillosis and asplenia.  **Suspected infection source and clinical course:** Urinary (for BSI), also diagnosed with respiratory tract infection due to Parainfluenza virus  **Antibiotics:** Empirical therapy with piperacillin-tazobactam and vancomycin for 1 day, then meropenem for 5 days + azithromycin for 3 days. Stepped down to oral ciprofloxacin to complete a 14-day course. | Extensive microbiological history, with isolation of *K. pneumoniae* multiple times from blood and urine, previous infection due to *Elizabethkingia anophelis*. | Likely false positive |

T2B = T2Bacteria result; BC = Blood Culture; F = Female; M = Male; ED = Emergency Department; ICU = Intensive Care Unit
